# Use of Selected Essential Oils to Control Aflatoxin Contaminated Stored Cashew and Detection of Aflatoxin Biosynthesis Gene

**DOI:** 10.1155/2015/958192

**Published:** 2015-01-18

**Authors:** Abeer R. M. Abd El-Aziz, Mohamed A. Mahmoud, Monira R. Al-Othman, Munirah F. Al-Gahtani

**Affiliations:** ^1^Botany and Microbiology Department, College of Science, King Saud University, Riyadh 1145, Saudi Arabia; ^2^Plant Pathology Research Institute, Agricultural Research Center, Giza 12619, Egypt

## Abstract

*Aspergillus* spp. associated with cashew from the regions of Riyadh, Dammam, and Abha were isolated and three different culture media were used to qualitatively measure aflatoxin production by *Aspergillus* via UV light (365 nm), which was expressed as positive or negative. The obtained data showed that six isolates of *A. flavus* and four isolates of *A. parasiticus* were positive for aflatoxin production, while all isolates of *A. niger* were negative. Five commercially essential oils (thyme, garlic, cinnamon, mint, and rosemary) were tested to determine their influence on growth and aflatoxin production in *A. flavus* and *A. parasiticus* by performing high-performance liquid chromatography (HPLC). The results showed that the tested essential oils caused highly significant inhibition of fungal growth and aflatoxin production in *A. flavus* and *A. parasiticus*. The extent of the inhibition of fungal growth and aflatoxin production was dependent on the type and concentration of essential oils applied. The results indicate that cinnamon and thyme oils show strong antimicrobial potential. PCR was used with four sets of primer pairs for *nor-1, omt-1, ver-1*, and *aflR* genes, enclosed in the aflatoxin biosynthetic pathway. The interpretation of the results revealed that PCR is a rapid and sensitive method.

## 1. Introduction

Cashew nut (*Anacardium occidentale *L.) is characterized by the high percentage of carbohydrates and lipids which facilitates attacking them by moulds, specifically* Penicillium *and* Aspergillus *[1].* Aspergillus flavus *and* Aspergillus parasiticus *are two of the most important toxigenic moulds [2]. The worldwide production of cashew is 4152315 (tonnes), area harvested (Ha) 5313415, and this crop yields 7815 (Hg/Ha) [3]. The aflatoxins are a group of chemically similar toxic fungal metabolites (mycotoxins) produced by genus* Aspergillus*. Aflatoxins are highly toxic compounds and can cause chronic toxicity in humans and animals. Aflatoxins may be present in a wide range of many food commodities, as nuts [4]. Many factors are influencing aflatoxin production during storage such as moisture, storage temperature, availability of oxygen, and lipids content [5]. There are four kinds of aflatoxins (B1, B2, G1, and G2) whereas aflatoxin B1 is the toxic kind. The presence of* Aspergillus* in stored cashew nuts appears in deterioration, discolouration, and bad odour [6]. Currently, synthetic chemicals are not used to manage mycotoxin production which cause harmful effects on consumers [7]. Recent trends toward natural product such as essential oil lead to safe and acceptable method. Many approaches investigated to manage aflatoxin production using essential oils [8–12].

The aims of the recent study were therefore to evaluate the effect of essential oils on the dry weight and aflatoxin production by* Aspergillus *spp. as well as to estimate the presence and absence of the PCR products corresponding to amplification of* aflD*,* aflM*,* aflP*,* aflR*, and* aflS* genes in aflatoxigenic and nonaflatoxigenic* A. flavus* isolates isolated from stored cashew.

## 2. Materials and Methods

### 2.1. Collection of Samples

Fifteen samples of cashew were collected randomly from different markets in three locations from Saudi Arabia (Riyadh, Dammam, and Abha) during 2012 for this experimental work. The samples were stored at 2°C until used [13].

### 2.2. Isolation, Purification, and Identification of Pathogen

Samples were surface sterilized with 5% sodium hypochlorite solution for one minute; then wash these samples three times with sterilized distilled water. Four pieces were placed on the surface of potato dextrose agar Petri dishes with three replicated times. Petri dishes were incubated at 25°C for 7 days. Isolates were purified by single spore methods. The identification of* Aspergillus flavus* and* A. parasiticus* isolates was confirmed by Regional Center of the Fungi and their Applications, Al-Azhar University, Cairo, Egypt.

### 2.3. Detection of Aflatoxin Production in Different Culture Media via Fluorescence (UV)

Three different media (potato dextrose agar (PDA), Czapek agar (CZ), and malt extract agar (MEA)) were used for detection of aflatoxin based on fluorescence [16]. Where the plates were incubated at 25°C for 4 days in the dark, the presence or absence of fluorescence in the agar surrounding the growing* Aspergillus *colonies was determined by exposing the Petri dishes to ultraviolet light (365 nm) and expressed as positive or negative according to [17].

### 2.4. Effect of Essential Oils on Growth and Aflatoxin Produced by* Aspergillus* spp

Antiaflatoxigenic efficacy values of each tested oil were determined using SMKY liquid medium (sucrose, 20 g; magnesium sulfate, 0.5 g; potassium nitrate, 3 g; yeast extract, 7 g; and distilled water, 1000 mL) [18]. The surfactant (25% Tween in sterile water) was added to oils; then different doses of each oil (1, 2, and 4%) were prepared and added to media; the flasks were inoculated with discs of 6 mm diameter of the toxigenic* Aspergillus* spp. at 25 ± 1°C for 7 days [7]. Three replicates were performed for each concentration and control was carried out with Tween 80 [19]. After incubation, content of each flask was filtered (Whatman, number 1) and biomass of filtered mycelium was dried at 70°C for 4 days till their weights remain constant. For aflatoxins extractions, the filtrates of each flask were treated three times with 50 mL of chloroform in a separating funnel then dehydrated with anhydrous sodium sulfate and evaporated on water bath at 50°C under vacuum. The residues were dissolved in 10 mL methanol [20].

### 2.5. Determination of Aflatoxins by High-Performance Liquid Chromatography (HPLC)

#### 2.5.1. Standard and Calibration Preparation

B1, B2, G1, and G2 (1 mg) aflatoxin standards were purchased from Sigma (St. Louis, MO, USA). The stock solutions (10 *μ*g mL^−1^) and working solutions (1 *μ*g mL^−1^) were prepared in methanol (HPLC), with assay >99.9% (Sigma).

#### 2.5.2. Sample Preparation

The sample was passed through a 0.45 *μ*m microfilter. Analysis of compounds was performed on HPLC model (model PerkinElmer series 200 UV/VIS) with a C18 column with an internal diameter of 300 mm × 3.9 mm, 4 micron. The HPLC was equipped with an UV detector and fluorescence with 365 nm excitation and 430 emission wavelengths. The mobile phase consists of methanol : acetic acid : water (20 : 20 : 60 v/v/v). The total run time for the separation was approximately 25 min at a flow rate of 1 mL/min [21]. The aflatoxin inhibition was calculated as follows:

(1) $$\begin{array}{c} \%\;\;\text{inhibition}=\left[\frac{A-a}{A}\right]\times 100, \end{array}$$

where “*A*” is aflatoxin in the control and “*a*” is aflatoxin in the treated sample.

### 2.6. Extraction of DNA from* Aspergillus* Isolates

*Aspergillus* isolates were cultured on double layer media in 50 mm Petri dishes, one solid and the other liquid. Base media, solid, was potato dextrose agar as a film, and the top media, liquid, was peptone yeast glucose (PYG, 1200 *μ*L) and was then incubated at 25°C for two days. Genomic DNA extraction was according to [22].

### 2.7. PCR Assay for Four Aflatoxins Biosynthesis Genes

PCR parameters followed those reported by [14]. PCR was performed in 25 *μ*L containing 2.5 *μ*L of 10X PCR buffer, 0.75 *μ*L of 25 mM MgCl_2_, 0.5 *μ*L of 10 mM dNTPs, 0.625 *μ*L of each primer, 5 U Taq DNA polymerase, 2 *μ*L of extracted DNA as template, and 17.5 *μ*L of sterile distilled water. A total of 35 PCR cycles with the following temperature regimen was performed: 95°C, 1 min; 65°C, 30 s; and 72°C, 30 s for the first cycle and 94°C, 30 s; 65°C, 30 s; and 72°C, 30 s for the next 34 cycles. Genes* aflD*,* aflM*,* aflP*, and* aflR* were tested in all isolates using the primer pairs listed in Table 1.

### 2.8. Statistical Analysis

All of the data from three independent replicate trials were subjected to analysis using Statistical Package for the Social Sciences (SPSS) 10.0 statistical software (Chicago, USA). The data are reported as the mean ± standard deviations, and significant differences between mean values were determined with Duncan's multiple range test (*P* < 0.05), followed by one-way ANOVA.

## 3. Results

### 3.1. Detection of Aflatoxins Produced by* Aspergillus* spp. Isolates from Cashews from Riyadh, Dammam, and Abha via UV Light

**Figure 1:**
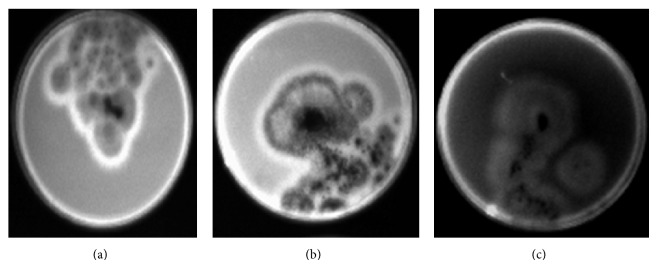
Aflatoxigenic strains of* A. flavus *(a),* A. parasiticus *(b), and* A. niger *(c) visualized under 365 nm UV light. The white ring around colonies of aflatoxigenic strains displays faint blue fluorescence.

Data in Table 2 show that three isolates of* A. niger*, two isolates of* A. parasiticus*, and six isolates of* A. flavus* were isolated from Riyadh and detected under UV light whereas three isolates (RC4, RC5, and RC10) of* Aspergillus* spp. were expressed as positive for aflatoxin production. In Dammam, four isolates of* A. niger*, two isolates of* A. parasiticus*, and six isolates of* A. flavus* were isolated and detected under UV light whereas four isolates (DC5, DC6, DC7, and DC8) of* Aspergillus* spp. were expressed as positive for aflatoxin production. In Abha, three isolates of* A. niger*, two isolates of* A. parasiticus*, and five isolates of* A. flavus* were isolated and detected under UV light whereas three isolates (AC2, AC4, and AC10) of* Aspergillus* spp. were expressed as positive for aflatoxin production. Example of non-aflatoxigenic and aflatoxigenic isolates are shown in Figure 1.

### 3.2. Effect of Five Essential Oils at Three Different Doses on Dry Weight of* A. flavus* and* A. parasiticus* Isolated from Cashews

Effect of different doses of essential oils on dry weight of mycelia after incubation at 25 ± 1°C for 7 days of* A. flavus *and* A. parasitica*. The all tested essential oils appeared more effective on the growth at three tested doses compared to control; % inhibition of dry weight decreased with increasing doses of all treatments by essential oils.

Tables 3 and 4 show that the highest growth inhibition rate of the tested fungi was isolated from cashews observed with the cinnamon oil and thyme oil at 4% in both* A. flavus *and* A. parasiticus* ranged from 50.4 to 72.7% and 44.2 to 70.1%, respectively. However, growth was slightly inhibited at the lower levels. Statistical results showed that kind and amount of essential oils have a significant influence on dry weight; *P* < 0.05.

### 3.3. Effect of Five Essential Oils at 4% on Aflatoxin B (*μ*g/mL) Produced by* A. flavus* That Were Isolated from Cashews

Data in Table 5 obtained that five tested essential oils that lead to a decrease in aflatoxins (B) were produced by* A. flavus* when compared with control. Thyme oil and cinnamon oil were the highest effective essential oils on inhibition of aflatoxin B which ranged from 53.5 to 86 and 59.2 to 78.7%, respectively. Mint oil gave the third rank followed by rosemary oil; inhibition ranged from 46.1 to 71.9 and 38.6 to 64.4%, respectively, whereas the least effective essential oil was garlic oil (ranged from 28.5 to 62.8%). The results indicate that the tested toxigenic fungi are sensitive to the tested five essential oils; particularly isolate number AC2 was more sensitive to any tested oils: thyme, cinnamon, mint, rosemary, and garlic (86.0, 78.5, 71.9, 64.4, and 62.8%, resp.), while the least of inhibition was observed in isolate number AC4 (28.5%) when treated with garlic oil.

### 3.4. Effect of Five Essential Oils at 4% on Aflatoxin G (*μ*g/mL) That Were Produced by* A. flavus* Isolated from Cashews

Data in Table 6 obtained that cinnamon oil was the highest effective essential oil on inhibition of aflatoxin G followed by thyme oil at 4% which leads to the highest level of inhibition ranging from 65.5 to 87.3 and 58.8 to 67.3%, respectively. Treatment with cinnamon oil leads to complete inhibition of aflatoxin G2 produced by isolates number AC4. Mint oil gave the third rank followed by Rosemary oil; inhibition ranged from 55.1 to 67.7 and 45.3 to 60.2%, respectively, whereas garlic oil gave the least (34.1 to 45.8%). In addition to that, the results indicate that the highest level of inhibition % was observed in isolates number AC2, while the least of inhibition was observed in isolates number DC6.

### 3.5. Effect of Five Essential Oils at 4% on Aflatoxin B (*μ*g/mL) Produced by* A. parasiticus* That Were Isolated from Cashews

Data in Table 7 obtained that five tested essential oils which lead to a decrease in aflatoxins (B) were produced by* A. parasiticus* when compared with control. Thyme oil and cinnamon oil were the highest effective essential oils on inhibition of aflatoxin B which ranged from 60.8 to 71.6 and 67.3 to 70.7%, respectively. Mint oil and rosemary oil gave the third rank; inhibition ranged from 52.9 to 57.7 and 48.7 to 59.8%, respectively, whereas garlic essential oil ranged from 40.8 to 47.9%. In addition to that, treatment with cinnamon oil leads to complete inhibition on production of aflatoxin B2 in two isolates numbers RC10 and DC5.

### 3.6. Effect of Five Essential Oils at 4% on Aflatoxin G (*μ*g/mL) That Were Produced by* A. parasiticus* Isolated from Cashews

Data in Table 8 obtained that cinnamon oil was the highest effective essential oil on inhibition of aflatoxin G followed by thyme oil at 4% which leads to the highest level of inhibition ranging from 57.1 to 70.8 and 52.4 to 61.2%, respectively. Rosemary oil gave the third rank followed by mint oil; inhibition ranged from 50.2 to 65.3 and 51 to 58.3%, respectively, whereas garlic oil gave the least (27.9 to 47.4%). In addition to that, the results indicate that the highest level of inhibition of aflatoxin G was observed in isolate number RC10 at most cases, while the least inhibition was observed in isolate number AC10 when treated with any tested essential oil except treatment with rosemary oil.

### 3.7. Detection of Four Aflatoxins Biosynthesis Genes

**Figure 2:**
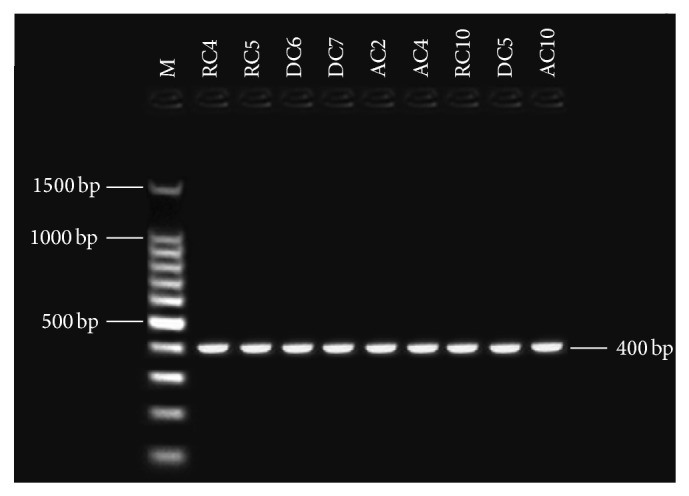
Gel electrophoresis analysis PCR products using* nor-1 *primer with 400 bp, lanes from RC4 to AC4 (*A. flavus*), lanes from RC10 to AC10 (*A. parasiticus*), and lane M: 100 bp DNA ladder size marker.

**Figure 3:**
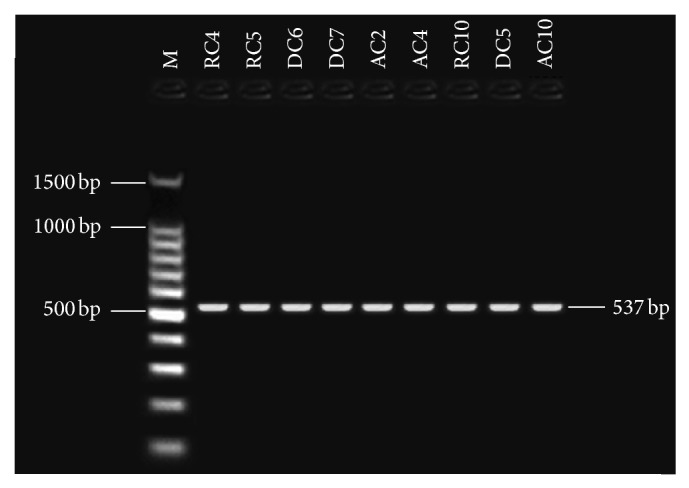
Gel electrophoresis analysis PCR products using* aflR* primer with 1032 bp, lanes from RC4 to AC4 (*A. flavus*), lanes from RC10 to AC10 (*A. parasiticus*), and lane M: 100 bp DNA ladder size marker.

**Figure 4:**
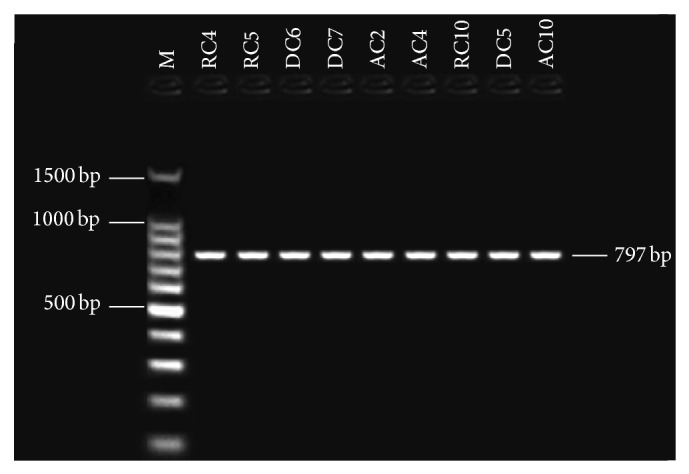
Gel electrophoresis analysis PCR products using* ver-1 *primer with 537 bp, lanes from RC4 to AC4 (*A. flavus*), lanes from RC10 to AC10 (*A. parasiticus*), and lane M: 100 bp DNA ladder size marker.

**Figure 5:**
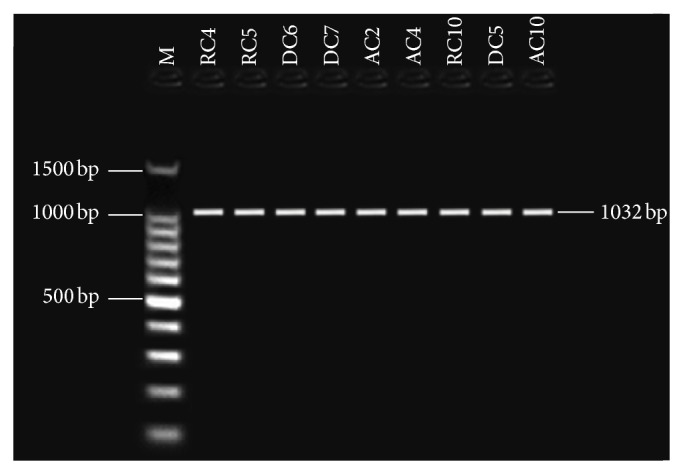
Gel electrophoresis analysis PCR products using* omt-1 *primer with 797 bp, lanes from RC4 to AC4 (*A. flavus*), lanes from RC10 to AC10 (*A. parasiticus*), and lane M: 100 bp DNA ladder size marker.

Primers pairs were used for this study to target four aflatoxin biosynthetic genes: the one regulatory gene* aflR* and the structural genes* nor-1* (*aflD*),* ver-1* (*aflM*), and* omt-1* (*aflP*). Detection of aflatoxin biosynthesis genes in nine aflatoxigenic* A. flavus* and* A. parasiticus* isolates. In PCR technique, the tested primer pairs were shown in the presence of the given amplified fragments for 9 aflatoxigenic* A. flavus *and* A. parasiticus* isolates. All aflatoxigenic isolates shown appear as single DNA fragment, indicating the presence of the gene. PCR was used with four sets of primer pairs for* nor-1*,* omt-1*,* ver-1*, and* aflR* genes, enclosed in the aflatoxin biosynthetic pathway. Bands of the fragments of* nor-1*,* ver-1*,* omt-1*, and* aflR* genes can be visualized at 400, 537, 797, and 1032 bp, respectively (Figures 2, 3, 4, and 5). The 67 isolates were analysed for the presence or absence of seven AF biosynthesis genes in relation to their ability to produce B1, targeting the two regulatory genes* aflR* and* aflS* and the five structural genes* aflD*,* aflM*,* aflO*,* aflP*, and* aflQ*. The isolates were divided into 4 groups based on their patterns of PCR products: group one (40 strains) distinguished by presence of all seven genes; groups two (two strains) and three (nine strains) showing four (*AflM*,* aflP*,* aflO*, and* aflQ*) and three (*aflO*,* aflP*, and* aflQ*) amplicons, respectively; and group IV (16 strains) distinguished by total absence of PCR products.

## 4. Discussion

Three culture media were used to screen examined of* Aspergillus* isolates for aflatoxin production (Czapek agar, potato dextrose agar, and yeast extract sucrose agar). The presence or absence of fluorescence in the agar surrounding the colonies assayed was determined under UV light (365 nm) [23]. Consequently,* Aspergillus flavus* and* A. parasiticus* were expressed as positive or negative, whereas all isolates of* A. niger* were expressed as negative. This result agrees with [24, 25]. Data show that the all tested essential oils (cinnamon, garlic, mint, rosemary, and thyme) appeared more effective on the growth at three tested doses compared to control. There is a direct relationship between the inhibitory effect of the oils and their doses [25–27].

Five tested essential oils that lead to a decrease in aflatoxins (B) were produced by* A. flavus* and* A. parasiticus* when compared with control. The results indicate that the test toxigenic fungi are sensitive to the 5 essential oils and particularly sensitive to thyme and cinnamon. This result is confirmed by many researchers [28–30].

The effect of essential oil might be due to aliphatic aldehydes and their ability to form charge transfer complexes with electron donors in the fungus cell [31]. Essential oil may be conducive to changes in the structure of the cells and denaturation of the enzymes which affects fungal morphogenesis and growth [32]. Essential oil leads to interference with the amino acid involved in germination [33]. Regional differences in aflatoxin contamination of crops may be attributable to climatic conditions and to agricultural practices that increase susceptibility of plants to invasion by* A. flavus* and relative humidity plays a vital role in the development and spread of fungal contaminations [34] and preharvest conditions of temperature and humidity in the field and improper postharvest handling and storage [35, 36].

Cluster genes in aflatoxin biosynthesis pathway contain structural genes,* nor-1*,* ver-1*, and* omt-1*, and* aflR* is a regulatory gene that plays a key role in the production of AF. The results indicated that three toxigenic isolates of* A. flavus* were positive using TLC technique and the appearing bands of the unique fragments of* aflR*,* omt-1*,* ver-1*, and* nor-1* genes can be visualized at 1032, 1232, 895, and 400 bp, respectively. Positive results were obtained only with extracts of* A. flavus*, even at the lowest spore concentration. The elucidation of the results revealed that specific PCR for AF biosynthesis genes is of a high sensitivity and is rapid (100%) [14, 37].

## Figures and Tables

**Table 1:** Details of the target genes, primer sequences, and expected product length in base pairs (bp) for PCR.

Primer pair	Gene	Primer sequence (5′ → 3′)	PCR product size (bp)	Reference
Nor-1F	*aflD* (*nor-1*)^a^	ACCGCTACGCCGGCACTCTCGGCAC	400	
Nor-1R		GTT GGCCGCCAGCTTCGACACTCCG		
Ver-1F	*aflM *(*ver-1*)	GCCGCAGGCCGCGGAGAAAGTGGT	537	
Ver-1R		GGGGATATACTCCCGCGACACAGCC		[14]
Omt-1F	*aflP *(*omt-1*)	GTGGACGGACCTAGTCCGACATCAC	797	
Omt-1R		GTCGGCGCCACGCACTGGGTTGGGG		
aflR-F	*aflR *	TATCTCCCCCCGGGCATCTCCCGG	1032	
aflR-R		GTCGGCGCCACGCACTGGGTTGGGG		

^a^Aflatoxin biosynthetic genes are named as proposed by [15]; old names are reported in brackets.

**Table 2:** Detection of aflatoxins produced by *Aspergillus* spp. isolates from cashews via UV light.

Region	Isolates code	*Aspergillus* spp.	Culture media		
			Yeast extract	Czapek-Dox agar	PDA + NaCl
Riyadh	RC1	*A. niger *	−	−	−
Riyadh	RC2	*A. niger *	−	−	−
Riyadh	RC3	*A. niger *	−	−	−
Riyadh	RC4	*A. flavus *	+	+	+
Riyadh	RC5	*A. flavus *	−	+	−
Riyadh	RC6	*A. flavus *	−	−	−
Riyadh	RC 7	*A. flavus *	−	−	−
Riyadh	RC8	*A. flavus *	−	−	−
Riyadh	RC9	*A. flavus *	−	−	−
Riyadh	RC10	*A. parasiticus *	+	+	+
Riyadh	RC11	*A. parasiticus *	−	−	−
Dammam	DC1	*A. niger *	−	−	−
Dammam	DC2	*A. niger *	−	−	−
Dammam	DC3	*A. niger *	−	−	−
Dammam	DC4	*A. niger *	−	−	−
Dammam	DC5	*A. parasiticus *	+	−	−
Dammam	DC6	*A. parasiticus *	−	+	−
Dammam	DC7	*A. flavus *	+	−	−
Dammam	DC8	*A. flavus *	+	−	−
Dammam	DC9	*A. flavus *	−	−	−
Dammam	DC10	*A. flavus *	−	−	−
Dammam	DC11	*A. flavus *	−	−	−
Dammam	DC12	*A. flavus *	−	−	−
Abha	AC1	*A. flavus *	−	−	−
Abha	AC2	*A. flavus *	+	−	−
Abha	AC3	*A. flavus *	−	−	−
Abha	AC4	*A. flavus *	+	−	−
Abha	AC5	*A. flavus *	−	−	−
Abha	AC6	*A. niger *	−	−	−
Abha	AC7	*A. niger *	−	−	−
Abha	AC8	*A. niger *	−	−	−
Abha	AC9	*A. parasiticus *	−	−	+
Abha	AC10	*A. parasiticus *	+	+	−

**Table 3:** Effect of five essential oils at three different doses on dry weight of mycelia (g) of *A. flavus* and *A. parasiticus* isolated from cashews.

Isolates code	Control	Cinnamon			Garlic			Mint			Rosemary			Thyme		
		1%	2%	4%	1%	2%	4%	1%	2%	4%	1%	2%	4%	1%	2%	4%
RC4	3.69^a^ ± 0.15	2.58^ab^ ± 0.11	1.87^e^ ± 0.10	1.07^b^ ± 0.06	3.11^b^ ± 0.11	2.91^b^ ± 0.21	2.19^a^ ± 0.10	2.85^b^ ± 0.08	1.91^d^ ± 0.21	1.60^cd^ ± 0.05	3.0^ab^ ± 0.11	2.01^b^ ± 0.25	1.71^d^ ± 0.11	2.68^b^ ± 0.15	1.71^bc^ ± 0.07	1.13^e^ ± 0.08
RC5	3.61^a^ ± 0.13	2.74^b^ ± 0.08	2.01^e^ ± 0.05	1.79^bc^ ± 0.15	3.33^b^ ± 0.14	2.45^d^ ± 0.22	2.11^a^ ± 0.15	2.91^b^ ± 0.05	2.17^b^ ± 0.19	1.47^cd^ ± 0.08	3.15^ab^ ± 0.12	2.49^b^ ± 0.21	1.95^d^ ± 0.13	2.84^b^ ± 0.13	1.85^bc^ ± 0.08	1.57^f^ ± 0.05
RC10	4.09^b^ ± 0.05	3.11^c^ ± 0.11	2.67^cd^ ± 0.10	1.51^bc^ ± 0.15	4.0^ab^ ± 0.15	3.19^ab^ ± 0.16	2.41^d^ ± 0.10	3.45^ab^ ± 0.06	2.81^bc^ ± 0.16	2.14^a^ ± 0.09	3.02^ab^ ± 0.15	2.76^a^ ± 0.23	1.75^d^ ± 0.10	2.87^b^ ± 0.12	2.45^d^ ± 0.05	1.22^e^ ± 0.11
DC5	2.87^d^ ± 0.08	1.88^a^ ± 0.15	1.78^bc^ ± 0.12	1.09^b^ ± 0.08	2.71^d^ ± 0.10	2.18^a^ ± 0.08	1.87^ab^ ± 0.15	2.06^d^ ± 0.07	1.87^e^ ± 0.17	1.72^cd^ ± 0.11	2.01^b^ ± 0.05	1.91^d^ ± 0.12	1.33^a^ ± 0.13	2.0^bc^ ± 0.17	1.63^bc^ ± 0.11	1.17^e^ ± 0.10
DC6	3.62^a^ ± 0.11	2.77^b^ ± 0.05	2.41^cd^ ± 0.10	1.45^bc^ ± 0.05	3.23^b^ ± 0.11	3.0^b^ ± 0.10	2.31^a^ ± 0.10	3.02^ab^ ± 0.13	2.80^bc^ ± 0.15	1.92^cd^ ± 0.18	2.93^ab^ ± 0.04	2.78^a^ ± 0.16	2.01^b^ ± 0.14	2.75^b^ ± 0.13	2.0^d^ ± 0.10	1.62^f^ ± 0.19
DC7	3.55^a^ ± 0.16	2.44^b^ ± 0.06	2.08^ab^ ± 0.22	1.11^b^ ± 0.15	3.0^b^ ± 0.13	2.79^b^ ± 0.17	2.45^a^ ± 0.12	3.0^ab^ ± 0.15	2.18^b^ ± 0.16	1.74^cd^ ± 0.15	3.05^ab^ ± 0.10	2.47^b^ ± 0.15	1.87^d^ ± 0.15	2.57^b^ ± 0.14	1.80^bc^ ± 0.08	1.42^f^ ± 0.11
DC8	4.12^b^ ± 0.06	2.68^ab^ ± 0.06	1.77^bc^ ± 0.12	1.28^abc^ ± 0.15	4.0^ab^ ± 0.15	3.10^ab^ ± 0.13	2.84^abc^ ± 0.10	2.85^bc^ ± 0.11	2.35^b^ ± 0.18	2.0^a^ ± 0.10	2.65^b^ ± 0.08	2.08^b^ ± 0.07	1.60^d^ ± 0.08	2.74^b^ ± 0.16	1.80^bc^ ± 0.10	1.36^f^ ± 0.13
AC2	3.94^b^ ± 0.12	2.88^ab^ ± 0.10	2.03^cd^ ± 0.17	1.88^bc^ ± 0.15	3.78^ab^ ± 0.09	3.0^b^ ± 0.17	2.81^abc^ ± 0.10	3.0^bc^ ± 0.12	2.80^bc^ ± 0.15	2.01^a^ ± 0.10	3.12^ab^ ± 0.11	2.29^b^ ± 0.09	1.89^d^ ± 0.11	2.91^bc^ ± 0.16	2.13^d^ ± 0.15	1.85^a^ ± 0.08
AC4	3.75^a^ ± 0.10	2.78^b^ ± 0.15	2.05^cd^ ± 0.10	1.19^b^ ± 0.05	3.22^b^ ± 0.08	3.0^b^ ± 0.16	2.75^abc^ ± 0.12	2.10^d^ ± 0.13	2.85^bc^ ± 0.20	2.64^ab^ ± 0.11	2.68 ± 0.06	2.10^b^ ± 0.08	1.78^d^ ± 0.12	2.98^bc^ ± 0.10	1.56^a^ ± 0.11	1.23^e^ ± 0.11
AC9	4.01^b^ ± 0.15	2.34^ab^ ± 0.10	2.01^cd^ ± 0.09	1.23 ± 0.05	3.54^ab^ ± 0.06	3.0^b^ ± 0.15	2.01^a^ ± 0.11	3.34^ab^ ± 0.10	2.55^bc^ ± 0.19	2.11^abc^ ± 0.12	3.07^ab^ ± 0.11	2.33^b^ ± 0.06	1.66^d^ ± 0.08	2.87^bc^ ± 0.09	1.68^bc^ ± 0.11	1.44^f^ ± 0.21
AC10	4.11^b^ ± 0.11	2.68^abc^ ± 0.09	1.98^e^ ± 0.16	1.12 ± 0.06	3.87^ab^ ± 0.05	3.12^ab^ ± 0.12	2.13^a^ ± 0.13	3.11^ab^ ± 0.15	2.75^bc^ ± 0.25	1.78^cd^ ± 0.13	3.18^ab^ ± 0.13	2.22^b^ ± 0.10	1.88^d^ ± 0.10	2.55^cd^ ± 0.14	1.93^bc^ ± 0.13	1.32^e^ ± 0.13
LSD0.05	0.332	0.412	0.314	0.256	0.516	0.423	0.263	0.412	0.365	0.511	0.612	0.432	0.279	0.416	0.307	0.214

Values in the same column followed by ± are significantly different (*P* = 0.05). The data shown are the means (*n* = 3) ± standard error of three replicates; data followed by the same letter are not significant at *P* ≤ 0.05, but data followed by different letters are significant at *P* ≤ 0.05.

**Table 4:** Effect of five essential oils at three different doses on % inhibition of dry weight of *A. flavus* and *A. parasiticus* isolated from cashews.

Isolates code	Cinnamon			Garlic			Mint			Rosemary			Thyme		
	1%	2%	4%	1%	2%	4%	1%	2%	4%	1%	2%	4%	1%	2%	4%
RC4	30.0	51.2	71.0	15.9	21.4	40.6	24.1	48.5	56.6	18.6	45.7	53.6	29.5	53.9	70.1
RC5	24.0	44.5	50.4	8.5	33.5	41.5	19.6	41.8	59.2	14.1	33.5	45.9	22.4	50.1	58.4
RC10	23.9	36.4	63.6	2.2	24.2	41.0	16.8	31.5	47.6	26.6	33.9	57.2	31.5	41.3	70.6
DC5	34.4	40.7	62.0	5.9	26.8	34.8	30.3	37.2	40.0	30.3	33.7	53.6	53.6	30.3	44.2
DC6	23.4	33.7	59.9	11.6	17.1	36.1	17.1	22.6	46.9	19.8	25.4	44.4	25.4	44.7	55.8
DC7	31.2	43.6	68.7	15.4	23.9	30.9	15.4	40.8	50.9	15.4	32.3	47.3	29.5	49.2	60.5
DC8	34.9	58.7	68.9	2.9	24.7	31.0	32.0	44.1	51.4	36.8	51.4	61.1	34.4	56.3	68.4
AC2	26.9	49.2	52.2	6.0	23.8	28.6	23.8	28.9	48.9	21.3	44.1	52.0	26.3	46.7	54.3
AC4	25.8	46.6	68.2	14.6	20.0	26.6	44.0	25.3	29.6	30.6	44.0	52.5	22.6	60.0	68.0
AC9	41.6	50.1	69.3	12.7	25.1	49.8	17.7	37.6	47.3	25.1	42.6	58.6	30.1	60.1	65.0
AC10	34.7	53.7	72.7	7.5	24.5	48.1	24.5	34.3	56.6	24.5	46.4	54.2	39.1	53.7	68.3

**Table 5:** Effect of five essential oils at 4% on aflatoxin B (*µ*g/mL) produced by *A. flavus* isolated cashews.

Isolates code	Control		Cinnamon		% inhibition	Garlic		% inhibition	Mint		% inhibition	Rosemary		% inhibition	Thyme		% inhibition
	B1	B2	B1	B2		B1	B2		B1	B2		B1	B2		B1	B2	
RC4	21.1	7.1	9.8	1.7	59.2	14.2	3.0	39.0	11.0	4.2	46.1	12.3	5.0	38.6	11.1	2.0	53.5
RC5	23.0	8.0	9.3	2.9	60.6	11.0	4.6	49.7	9.7	3.3	58.1	10.2	4.2	53.5	8.1	2.3	66.4
DC6	52.4	10.8	21.1	3.3	61.4	29.5	5.3	44.9	23.5	4.1	56.3	26.2	5.0	50.6	15.2	1.8	73.1
DC7	34.0	10.7	11.8	4.1	64.4	14.3	6.2	54.1	10.5	5.1	65.1	13.3	4.0	61.2	8.9	1.4	64.4
DC8	0	0	0	0	0	0	0	0	0	0	0	0	0	0	0	0	0
AC2	6.9	5.2	2.6	0	78.5	3.1	1.4	62.8	2.4	1.0	71.9	1.9	2.4	64.4	0	1.7	86.0
AC4	10.7	3.7	3.9	1.1	65.3	8.0	2.3	28.5	4.3	1.1	62.5	6.1	0.7	52.7	4.4	0.81	63.8

**Table 6:** Effect of five essential oils at 4% on aflatoxin G (*µ*g/mL) produced by *A. flavus* isolated from cashews.

Isolates code	Control		Cinnamon		% inhibition	Garlic		% inhibition	Mint		% inhibition	Rosemary		% inhibition	Thyme		% inhibition
	G1	G2	G1	G2		G1	G2		G1	G2		G1	G2		G1	G2	
RC4	34.9	9.5	11.6	2.7	67.8	20.3	6.2	40.3	14.1	5.0	57.0	16.0	3.0	57.2	12.3	4.1	63.1
RC5	0	0	0	0	0	0	0	0	0	0	0	0	0	0	0	0	0
DC6	0	26.7	0	9.2	65.5	0	17.6	34.1	0	12.0	55.1	0	14.1	47.1	0	11.0	58.8
DC7	29.5	0	6.1	0	79.3	16.0	0	45.8	12.1	0	59.0	12.9	0	56.3	10.3	0	65.1
DC8	0	0	0	0	0	0	0	0	0	0	0	0	0	0	0	0	0
AC2	25.1	0	3.2	0	87.3	15.3	0	39.0	8.1	0	67.7	10.0	0	60.2	8.2	0	67.3
AC4	17.4	5.8	3.2	0.0	86.2	9.1	4.0	43.5	7.5	1.8	60.0	8.3	4.4	45.3	6.1	2.7	60.1

**Table 7:** Effect of five essential oils at 4% on aflatoxin B (*μ*g/mL) produced by *A. parasiticus* isolated cashews.

Isolates code	Control		Cinnamon		% inhibition	Garlic		% inhibition	Mint		% inhibition	Rosemary		% inhibition	Thyme		% inhibition
	B1	B2	B1	B2		B1	B2		B1	B2		B1	B2		B1	B2	
RC10	28.9	5.6	10.1	0	70.7	16.2	4.2	40.8	14.4	1.8	53.0	15.1	2.6	48.7	12.3	1.2	60.8
DC5	39.2	5.2	13.5	0	69.6	20.1	3.7	46.4	15.5	3.5	57.7	17.3	3.1	54.1	10.0	2.6	71.6
AC10	7.0	4.7	3.1	0.73	67.3	4.3	1.8	47.9	3.7	1.8	52.9	3.1	1.6	59.8	3.2	1.3	61.5

**Table 8:** Effect of five essential oils at 4% on aflatoxin G (*μ*g/mL) produced by *A. parasiticus* isolated from cashews.

Isolates code	Control		Cinnamon		% inhibition	Garlic		% inhibition	Mint		% inhibition	Rosemary		% inhibition	Thyme		% inhibition
	G1	G2	G1	G2		G1	G2		G1	G2		G1	G2		G1	G2	
RC10	19.3	10.2	6.2	2.4	70.8	10.4	5.1	47.4	8.0	4.3	58.3	8.3	3.8	58.9	6.7	4.7	61.2
DC5	61.5	0	23.2	0	62.3	34.2	0	44.3	28.3	0	53.9	30.6	0	50.2	26.3	0	57.2
AC10	14.7	0	6.3	0	57.1	8.6	0	27.9	7.2	0	51.0	5.1	0	65.3	7.0	0	52.4
